# Histological analysis of tonsillectomy and adenoidectomy specimens - January 2001 to May 2003

**DOI:** 10.1016/S1808-8694(15)31279-9

**Published:** 2015-10-20

**Authors:** Alfredo R. Dell'Aringa, Antônio J.C. Juares, Cinthia de Melo, José C. Nardi, Kazue Kobari, Renato M. Perches Filho

**Affiliations:** 1 Ph.D. and Head of the Discipline of Otorhinolaryngology, Medical School, Marília; 2 Resident Physician in Otorhinolaryngology; 3 Resident Physician in Otorhinolaryngology; 4 Master and Professor, Discipline of Otorhinolaryngology; 5 Professor, Discipline of Otorhinolaryngology; 6 Resident Physician in Otorhinolaryngology

**Keywords:** adenotonsilectomia, histopatológica, adenotonsilectomy, hispathologic

## Abstract

Palatine and nasopharyngeal tonsils are nonencapsulated nodular masses of lymphoid tissue of the respiratory and digestive tract epithelium.

**Study design:**

Retrospective clinical study based on the revision of medical records of patients who underwent tonsillectomy and adenoidectomy at Hospital das Clínicas, Medical School, Marília in the period between January 2001 and May 2003.

**Aim:**

Analysis of patients' profile and main pathological changes in 250 patients with palatine and nasopharyngeal tonsil hypertrophy, recurrent infections or both.

**Material and Method:**

Histological review of 250 patients who underwent tonsillectomy and adenoidectomy among adults and children.

**Results:**

Out of 250 subjects, 117 (46.8%) were female and 133 (53.2%) were male patients. Mean age was 7.3 years, ranging from 2 to 34 years. Main surgical indication was concomitant presence of recurrent infections and hypertrophy of nasopharyngeal and palatine tonsils. Among these patients, 160 (64%) were classified as grades III to IV hypertrophy. Lymphatic or follicular lymphatic hyperplasia was observed in 205 patients (82%); focal inflammation was verified in 45 (18%) subjects. Among those, 2 patients presented squamous cell cysts, 2 had Actinomyces sp colonies and 1 cat scratch disease. **Discussion**: The results presented in this study suggested a possible correlation between recurrent tonsillitis and palatine tonsil hypertrophy.

**Conclusion:**

Routine histological study of tonsillectomy and adenoidectomy specimens has a low cost-benefit rate, although, due to legal and ethical issues, physicians may request this type of examination.

## INTRODUCTION

Palatine and nasopharyngeal tonsils are lymphatic flesh clusters of tissue of the respiratory and digestive tract's epithelium. Classification by site includes palatine, lingual and pharyngeal tonsils. They have important role in the body's immune system, once they are near the entrance to the breathing passages where incoming antigens may be aspirated or ingested^1^, ^2^.

The palatine tonsils are the lateral walls that compose the bulk of the so-called Waldeyer's ring of lymphatic tissue^1^. They are coated by non-keratinous stratified epithelium, as an extension of the oral pharyngeal tissue, including 30 deep crypts that invaginate into the parenchyma, in which lymph nodes are found with the germ centers responsible for B-lymphocyte production^1^, ^3^. In addition to nodes, debris of epithelial cells desquamations, alive and dead lymphocytes, as well as bacterium may be present in the crypts. In cases of acute tonsillitis, collection of pus may be observed^4^. They are coated by fibrous and dense capsule, separating them from a deeper connective tissue^1^, ^2^. This type of tonsils originates from the second pair of pharyngeal pouches, where endodermis bears the covering epithelium and mesenchymal structure gives origin to the lymphoid tissue^5^.

Pharyngeal tonsil (Luschka's tonsil) is a single entity and is located in the upper posterior pharynx; it consists of flat longitudinal folds, with sero-mucous glandular ducts opening on the base. They do not present crypts and their capsule is finer and incomplete^1^. They are covered by two types of epithelium: ciliated columnar pseudo-stratified with goblet cells and non-keratinous stratified epithelium. This may appear alone or as an associated condition, the latter being the most frequent^1^, ^2^, ^6^. They originate from lymph node clusters in the nasal-pharyngeal wall^5^.

Lingual tonsils are located within the base of the tongue and are formed by numerous and small structures. They are similar to palatine tonsils, however they do not present capsular involvement^1^.

During life, palatine and tongue tonsils may go through morphological alterations, becoming enlarged due to lymph follicles of the germ center; or histological changes resulting from recurrent infections, among which some are indicative of tonsillectomy^4^, ^7^.

According to Endo et al., it is general consensus that routine histological analysis of the removed tissues be performed, although otolaryngologists not always agree^8^. Our center usually performs histological analysis of tonsillectomy and adenoidectomy specimens. Lymphoid hyperplasia has shown to be the main histological alteration, although tonsils are not uncommon sites for onset of head and neck neoplasias. Regarding the above, 25% are benign conditions, such as squamous papillomas, lymphagiomas and squamous cell cysts. Among the malignant types, squamous cell carcinomas, lymphomas and other lymph-epithelial carcinomas are found^3^.

The purpose of this study was to report patients' profiles and the main histological changes found in 250 consecutive patients assisted at the Ambulatory of Otolaryngology of Medical School, ***Marília***, with enlarged palatine and pharyngeal tonsils, recurrent infections or both. Moreover, the present study aimed at analyzing histological findings, as well as the usefulness and cost-benefit of this evaluation as a routine procedure.

In this study, histological results of tonsillectomy and adenoidectomy specimens of patients with diagnoses of recurrent infections and/or obstructive hyperplasia in the airways were revised in the period of January 2001 and May 2003. Costs, and more specifically, cost-benefit rate of these histological tests adopted as a routine procedure were also analyzed.

## METHODOLOGY

This was a retrospective study based on data obtained from medical records of 250 patients who had undergone tonsillectomy or adenoidectomy in the period from January 2001 to May 2003 at the Department of Otolaryngology, Hospital das Clínicas, Medical School, Marília.

**Figure 1:**
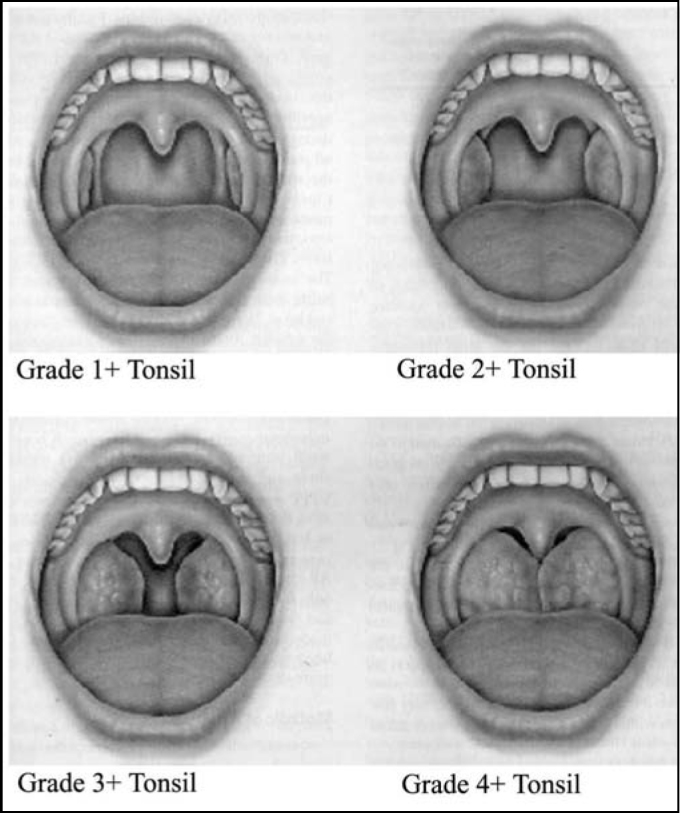
Grading of palatine tonsils hypertrophy proposed by L. Brodsky.

History of hypertrophic palatine and pharyngeal tonsils and/or recurrent tonsillitis was considered to select patients for assessment at the ambulatory. Palatine tonsils were classified according to the protocol proposed by L. Brodsky, shown on Figure 1. According to these scheme, it was considered that: 0 – tonsils inside the tonsillar fossa with no air obstruction; 1+ - tonsils slightly out of the tonsillar fossa presenting 25% air obstruction; 2+ - tonsils presenting 25-50% air obstruction; 3+ - tonsils presenting 50-75% air obstruction; 4+ - tonsils presenting 75% air obstruction^1º^.

All patients were submitted to general anesthesia and arch-form incision of the anterior pillar and peritonsillar dissection. Pharyngeal tonsils were removed with Beckmann curette. Patients underwent palatine and/or pharyngeal tonsillectomy as a result of clinical indication. Specimens were immediately stored in sterile glasses with 10% formaldehyde and sent for histological analysis at the Pathology Department, Medical School, ***Marília***^9^.

The surgical specimen was preserved in formol for 24 hours and, after dehydration, an histological procedure “en bloc” (5 μm thick) was done in paraffin, which was dyed in Hematoxillin-Eosin and analyzed microscopically^10^. The examinations followed the compliance standards and were conducted by pathologists of the Pathology Department.

## RESULTS

In this study, medical records of 250 patients submitted to tonsillectomy or adenoidectomy were revised.

**Figure 2:**
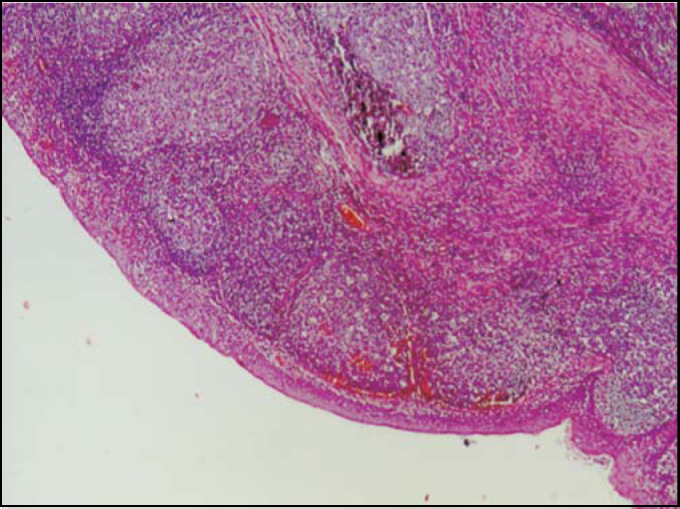
Normal lymphoid tissue clinical pathology analysis.

**Figure 3:**
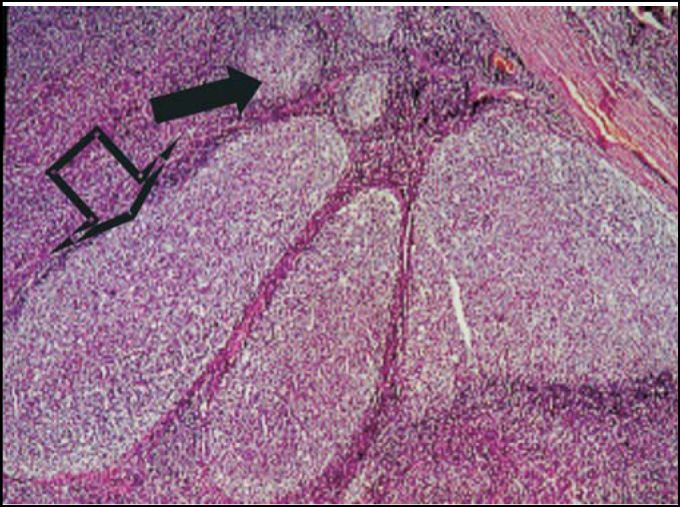
Follicular lymphoid hyperplasia. The solid arrow = normal follicles, and hollow arrow = no hyperplasic follicles.

**Figure 4:**
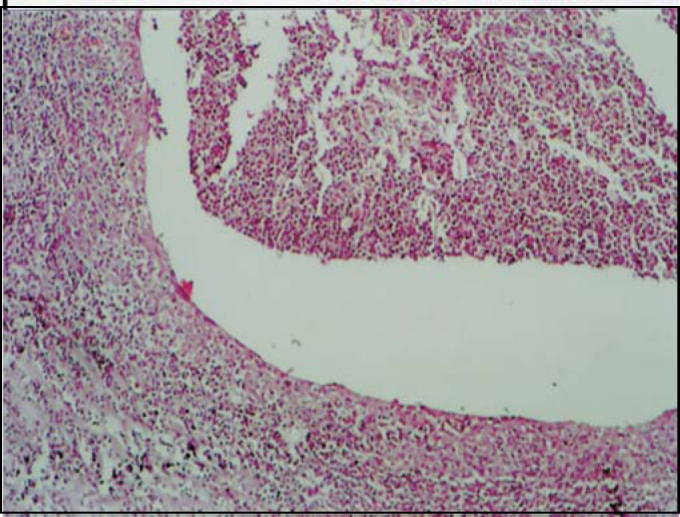
Local suppurative follicular lymphoid hyperplasia.

**Figure 5:**
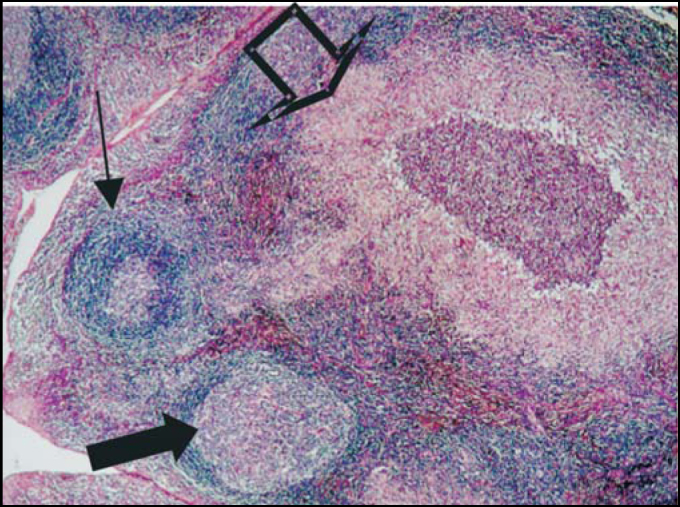
Granulomatous damage suggestive of cat's scratch disease. Solid arrow = hyperplasic follicle, hollow arrow = granuloma, and small arrow = normal follicle.

Among those, 117 (46.8%) were women and 133 (53.2%) were men. Patients' age ranged from 2 to 34 years, with mean age of 7.3 years. There were 236 patients (94.4%) younger than 18 years. Only 14 subjects (5.6%) were between 18 and 34 years of age.

Two hundred and five (205) tonsil-adenoidectomies and 45 tonsillectomies were performed. Indications were mainly due to hypertrophy of palatine and pharyngeal tonsils plus recurrent infections (174 cases – 69.6%); recurrent infections (12 cases – 4.8%) or hypertrophy only (64 cases – 25%). Subjects presenting enlarged tonsils were classified into 4 groups according to size: I (1+): 9 (3.6%); II (2+): 45 (18%); III (3+): 160 (64%); IV (4+): 26 (10.4%). Additional 10 patients presented asymmetric palatine tonsil, with predominance of grades II(2+) e III(3+) tonsils^11^. For pharyngeal tonsils evaluation, patient underwent paranasal sinuses radiography and/or nasofibroscopy; surgery indication was made only in case of hypertrophy.

Regarding the pathological examinations of palatine and pharyngeal tonsils, 143 patients (57.2%) presented lymphoid hyperplasia; 62 (24.8%) follicular lymphoid hyperplasia; and 45 (18%) suppurative focal acute inflammation; 2 patients had squamous cell cysts, 2 had small granules with Actinomyces sp colonies within the palatine tonsils' crypts and 1 had necrotic suppurative and granulomatous inflammation with small abscess full of neutrophils covered by a granulomatous inflammatory reaction, resembling a venereal lymphogranuloma. No cases of malignancy were verified among these patients.

## DISCUSSION

Among 250 patients, a slight predominance of men (53.2%) over women (46.8%) was observed. The majority of the cases were children (94.4%) presenting recurrent tonsillitis associated with hypertrophy of palatine and pharyngeal tonsils (69.6%), which is in accordance with the literature worldwide^3^, which may be consistent, considering that microorganisms growth could stimulate proliferation of lymphoid elements^12^. Only 9 patients (3.6%) had grade I tonsils, although presenting recurrent tonsillitis.

In the literature, inflammatory lesion of palatine tonsil crypts was observed with values between 10 and 37.66%^4^, ^13^ out of the total of patients. In this study, 18% of the subjects were found to have follicular lymphoid hyperplasia with acute focal suppurative inflammatory lesion in tonsil crypts.

Only 2 patients (0.8%) presented infections by Actinomyces sp colonies leading to tonsil hypertrophy and inflammatory lesion of palatine tonsil crypts. This incidence is significantly lower than that reported by Bhargava et al. in 2001 and Pransky et al. in 1991, who observed this condition in 8.5% of the patients with predominance of hypertrophy of palatine and pharyngeal tonsils over tonsillitis^14^, ^15^. However, our data are consistent with those reported by Sánchez et al. in 2006^16^. This low rate suggests that the presence of Actinomyces might not be related to hypertrophy of palatine and pharyngeal tonsils in these patients or, as Actinomyces is a common agent in the tonsillar tissue, it may not be routinely analyzed in the pathological specimens^8^.

Cat's scratch disease usually presents benign regional lymphadenitis in 50% of the patients with cervical tumors, which most of the times regress without complications or sequelae^17^. A particular patient in our study did not present cervical lymphadenitis nor had clinical complaints, rather presenting a good evolution and no need for antibiotic therapy. Surgery was performed due to airways obstruction.

The pathological analysis revealed the presence of squamous cell cysts in 2 patients, which are considered benign neoplasias in patients with recurrent tonsillitis. No malignant neoplasia was found, which is probably due to the low percentage of patients under the age of 18 years submitted to surgery, resulting in low incidence of these lesions.

Clinical analysis of specimen cost R13.89, while specimen from palatine and pharyngeal tonsillectomy together cost approximately R42.00. The findings included only presence of Actinomyces (0.8%), which is effectively resolved by surgery, according to Sánchez^11^; and a suspected Cat's Scratch disease, as mentioned above, which did not show signs of malignancy in the specimens examined.

## CONCLUSION

The above data led to the conclusion that hypertrophy of the palatine and pharyngeal tonsils may be associated with recurrent tonsillitis, as reported by most authors.

Classification of palatine tonsils hypertrophy, although subjective, proved to be effective in grading these conditions, once the result of the clinical pathology exams were mostly lymphoid hypertrophies - 82% of the patients.

Infiltration of Actinomyces sp. colonies and palatine tonsils hypertrophy do not seem to be related.

According to other authors, we agree that routine clinical pathology exams of tonsillectomy specimens in children present low cost-benefit ratio. However, due to legal and ethical reasons, physician may request this analysis.
